# Impact of a Liver Immune Status Index among Living Liver Transplant Recipients with Hepatocellular Carcinoma

**DOI:** 10.31662/jmaj.2023-0195

**Published:** 2024-02-27

**Authors:** Yuki Imaoka, Masahiro Ohira, Saki Sato, Ichiya Chogahara, Tomoaki Bekki, Kouki Imaoka, Ryosuke Nakano, Takuya Yano, Hiroshi Sakai, Shintaro Kuroda, Hiroyuki Tahara, Kentaro Ide, Tsuyoshi Kobayashi, Yuka Tanaka, Miho Akabane, Kazunari Sasaki, Hideki Ohdan

**Affiliations:** 1 Department of Gastroenterological and Transplant Surgery, Graduate School of Biomedical and Health Sciences Hiroshima University, Hiroshima University, Hiroshima, Japan; 2 Division of Regeneration and Medicine, Medical Center for Translational and Clinical Research, Hiroshima University Hospital, Hiroshima, Japan; 3 Division of Abdominal Transplant, Stanford University School of Medicine, Stanford, USA

**Keywords:** living-donor liver transplantation, hepatocellular carcinoma (HCC), natural killer (NK) cells

## Abstract

**Introduction:**

Hepatocellular carcinoma (HCC) is a major global health challenge, being the fifth most prevalent neoplasm and the third leading cause of cancer-related deaths worldwide. Liver transplantation offers a potentially curative approach for HCC, yet the risk of recurrence posttransplantation remains a significant concern. This study investigates the influence of a liver immune status index (LISI) on the prognosis of patients undergoing living-donor liver transplantation for HCC.

**Methods:**

In a single-center study spanning from 2001 to 2020, 113 patients undergoing living-donor liver transplantation for HCC were analyzed. LISI was calculated for each donor liver using body mass index, serum albumin levels, and the fibrosis-4 index. This study assessed the impact of donor LISI on short-term recurrence rates and survival, with special attention to its correlation with the antitumor activity of natural killer (NK) cells in the liver.

**Results:**

The patients were divided into two grades (high donor LISI, >−1.23 [n = 43]; and low donor LISI, ≤−1.23 [n = 70]). After propensity matching to adjust the background of recipient factors, the survival rates at 1 and 3 years were 92.6% and 88.9% and 81.5% and 70.4% in the low and high donor LISI groups, respectively (p = 0.11). The 1- and 3-year recurrence-free survival were 88.9% and 85.2% and 74.1% and 55.1% in the low and high donor LISI groups, respectively (p = 0.02).

**Conclusions:**

This study underscores the potential of an LISI as a noninvasive biomarker for assessing liver NK cell antitumor capacity, with implications for living-donor liver transplantation for HCC. Donor LISI emerges as a significant predictor of early recurrence risk following living-donor liver transplantation for HCC, highlighting the role of the liver antitumor activity of liver NK cells in managing liver malignancies.

## Introduction

Hepatocellular carcinoma (HCC) is the fifth most prevalent neoplasm and the third most frequent cause of cancer-related deaths in the world ^(1)^. Liver transplantation (LT) is recognized as a potential curative intervention for HCC. Theoretically, it represents the sole modality capable of eradicating tumor cells from the liver of the afflicted individual. However, in clinical practice, it is known that the recurrence of HCC post-LT ranges from approximately 10% to 20% ^(2)^.

Extant literature has underscored the significant correlation between tumor characteristics and the propensity for recurrence. Criteria such as the Barcelona Clinic Liver Cancer classification ^(3)^, the Milan criteria (tumor size ≤5 or ≤3 cm and tumor numbers ≤3) ^(4)^, 5-5-500 Japan criteria (tumor size ≤5 cm, tumor numbers ≤5, and α-fetoprotein (AFP) ≤500 ng/mL) ^(5)^, and HALT-HCC score (calculated from tumor sizes, tumor numbers, AFP levels, and MELD-Na scores) ^(6)^ have been established, predicated on tumor markers, the number of tumor lesions, and their respective diameters.

Our research has concentrated on the role of antitumor immunity within the hepatic environment, with a specific focus on the antineoplastic capabilities of natural killer (NK) cells in the liver. Through the analysis of liver NK cells sourced from donors of liver transplants, notable interindividual variances have been observed. Consequently, we have examined 40 living liver transplant donors, culminating in the development of a liver immune status index (LISI) ^(7)^, a metric designed to prognosticate the antitumor efficacy of liver NK cells.

The primary objective of this study was to evaluate the influence of the LISI, derived from donor livers, on the prognostic short-term outcomes of patients undergoing living-donor LT (LDLT) for HCC.

## Materials and Methods

### Patients

The LDLT for HCC was performed in Hiroshima University Hospital, Japan, as a single-institution study between 2001 and 2020. The patients’ clinical demographic data at the time of LT were obtained from the electronic records. The rates of HCC recurrence and survival after LT were also obtained from the clinical records. Patients were followed up using ultrasonography, contrast-enhanced computed tomography, or magnetic resonance imaging combined with an evaluation of serum AFP levels and des-γ carboxyprothrombin (DCP) at 3-, 6-, and 12-month intervals for up to 3 and 5 years and after 5 years, respectively. The diagnosis was histologically confirmed when necessary. Considering the anticipated limited temporal influence of donor factors, the outcomes selected for evaluation were confined to shorter time frames, specifically focusing on the 3-year overall survival (OS), recurrence-free survival (RFS), and recurrence rate (RR).

### Assessment of markers

The fibrosis-4 (FIB-4) index, initially developed for patients with human immunodeficiency virus and hepatitis C coinfection, was calculated using the following formula ^(8)^: (age [years] × aspartate aminotransferase [U/L]/(platelet count [10^9^/L] × √alanine aminotransferase [U/L]). LISI was calculated using the following formula: 36.39 − (6.18 × ALB) − {0.50 × body mass index (BMI)} + (2.91 × FIB-4 index) ^(7)^.

An inverse correlation exists between LISI and the expression of the antitumor molecule TNF-related apoptosis-inducing ligand (TRAIL) on liver NK cells. This suggests that elevated LISI levels are associated with a decrease in the antitumor efficacy of liver NK cells. The thresholds for high and low donor LISI categories in recipients were established using quartile values of donor LISI distribution, specifically using the receiver operating characteristic to predict the 3-year RFS. The patients were divided into two grades (high donor LISI, >−1.23 [n = 43]; and low donor LISI, ≤−1.23 [n = 70]).

### Adoptive NK cell therapy

This research also included a cohort of 42 individuals who underwent adoptive NK cell therapy, aimed at mitigating the recurrence of HCC and hepatitis C virus infection. This therapeutic approach, using activated liver allograft-derived lymphocytes, was approved by the Clinical Institutional Ethical Review Board of Hiroshima University (approvals Rin 414-1 and 40 019). Additionally, it has been registered with the University Hospital Medical Information Network under the identifiers 000012162 and 000000538, marking its status as a phase I clinical trial. The protocol for adoptive immunotherapy, which has been detailed in previous publications ^(9), (10)^, involves a series of intricate steps. Initially, liver mononuclear cells (LMNCs) are extracted from the effluent perfusates of liver allografts using gradient centrifugation. These LMNCs are then cultured in the presence of human recombinant interleukin-2 at a concentration of 100 Japanese reference units/mL (Celeuk, Takeda). This process continues for 3 days. Additionally, 12 h before infusion, either an OKT3 (at a concentration of 1 μg/mL, provided by Janssen Pharmaceutical K.K.) or anti-CD3 monoclonal antibody (at 1 μg/mL; MACS GMP CD3 pure, produced by Miltenyi Biotec K.K.) is introduced into the culture medium. This step is critical for targeting and opsonizing the CD3+ cell fraction. Three days after LT, the resultant activated liver NK cell-enriched lymphocytes are administered intravenously, thus completing the treatment protocol.

### Statistical analysis

The nonparametric Mann-Whitney U-test was performed to compare differences between the two independent groups; values of p < 0.05 were considered statistically signiﬁcant. Values are expressed as median [Q2-Q3]. The long-term survival was estimated and compared using the Kaplan-Meier and log-rank statistics. To adjust for differences in baseline characteristics, one-to-one propensity score models were constructed on the basis of each patient’s estimated propensity score based on the recipient factors, including tumor markers and pathological findings. Statistical analyses, including propensity score matching, were performed using JMP statistical software (JMP^Ⓡ^ 17; SAS Institute Inc., Cary, NC, USA). Values of p < 0.05 were considered statistically significant.

## Results

### Background of patients

In this investigation, a cohort of 113 initial LDLTs was examined, comprising 83 men (73.5%) and 30 women (26.5%). The study deliberately excluded cases of retransplantation and those involving deceased-donor LT (DDLT). The median age of the patients was established at 58 years, with an interquartile range (IQR) of 53-64 years. The median total tumor size was determined to be 20 mm, with an IQR of 13-29 mm. The median alpha-fetoprotein (AFP) level was recorded at 10.6 IU/mL, with an IQR of 5-40 IU/mL, and the median tumor count was 2, ranging from 1 to 4. As for the donor demographics, the median age was 34 years (IQR of 27-47 years), and the median donor LISI was calculated to be −1.94, with an IQR of −3.58 to −0.14. These demographic and clinical characteristics are comprehensively delineated in Table 1. The high donor LISI group had a significantly lower proportion of NK cell therapy and DCP levels compared with the high donor LISI group.

**Table 1.** Background Data According to Donor LISI Levels before Propensity Matching.

	High donor LISI (−1.23<)	Low donor LISI (≤−1.23)	p-value
Subject	N = 43	N = 70	
**Recipient factors**
Gender, male	35 (81.4)	48 (68.6)	0.13
Age (years)	56 [51-62]	58 [54-65]	0.08
BMI (kg/m^2^)	24.0 [21.7-26.4]	24.5 [21.6-27.0]	0.37
GWRW	0.86 [0.76-1.0]	0.85 [0.75-1.0]	0.88
Outside the Milan criteria	18 (41.9)	34 (48.6)	0.48
Outside the Japan criteria	6 (14.0)	12 (17.1)	0.65
MELD score	12 [8.3-16]	13 [10.3-18]	0.29
Cold ischemic time (min)	90 [66-112]	78 [51-115]	0.08
Fibrosis-4 index	0.66 [0.47-1.12]	0.89 [0.63-1.14]	0.10
Recipient LISI	7.64 [4.47-10.0]	8.21 [5.10-11.22]	0.17
NK cell therapy	10 (23.3)	32 (45.7)	0.02
Operation time (min)	723 [670-785]	753 [663-844]	0.17
Bleeding volume (mL)	2,970 [2,100-4,900]	3,695 [2,216-5,611]	0.27
AFP (ng/mL)	13.4 [5.6-50.9]	8.1 [5-40.0]	0.29
DCP (mAU/mL)	27 [15-94]	78 [25-519]	<0.01
Timor size (mm)	20 [14-28]	20 [12-30]	0.75
Tumor number	2 [1-4]	2 [1-4]	0.73
Vascular invasion, yes	11 (25.6)	12 (17.1)	0.28
Poorly differentiated, yes	4 (9.3)	8 (11.4)	0.72
**Donor factors**
Gender, male	20 (46.5)	53 (75.7)	<0.01
Age (years)	43 [29-54]	30.5 [27-38]	<0.01
BMI (kg/m^2^)	20.7 [19.7-21.9]	22.9 [20.5-24.5]	<0.01
Donor fibrosis-4 index	0.86 [0.59-1.16]	0.54 [0.41-0.75]	<0.01
Donor LISI	0.70 [−0.53-2.22]	−3.15 [−4.2-2.22]	<0.01

BMI, body mass index; GWRW, graft weight-recipient weight ratio; NK, natural killer; AFP, α-fetoprotein; DCP, des-γ carboxyprothrombin; LISI, liver immune status index

To minimize the influence of recipient tumor characteristics on the study outcomes, a propensity score matching approach was used. This methodological adjustment was aimed at ensuring a balanced comparison between the two groups, particularly in terms of tumor marker levels and pathological tumor findings. The postmatching demographic and clinical characteristics of the patients are succinctly summarized in Table 2, providing a detailed and comparative analysis of the study population. After propensity score matching, there were no significant differences in NK cell therapy and DCP levels between the two groups.

**Table 2.** Background Data According to Donor LISI Levels after Propensity Matching.

	High donor LISI (−1.23<)	Low donor LISI (≤−1.23)	p-value
Subject	N = 27	N = 27	
**Recipient factors**
Gender, male	21 (77.8)	19 (70.4)	0.53
Age (years)	56 [51-61]	56 [53-60]	0.84
BMI (kg/m^2^)	23.7 [21.7-26.4]	23.6 [21.4-27.3]	0.80
GWRW	0.84 [0.75-0.90]	0.85 [0.75-0.99]	0.42
Outside the Milan criteria	11 (40.7)	10 (37.0)	0.78
Outside the Japan criteria	4 (14.8)	3 (11.1)	0.69
MELD score	12 [9-17]	12 [10-14]	0.81
Cold ischemic time (min)	89 [57-123]	92 [70-119]	0.78
Fibrosis-4 index	0.69 [0.49-1.12]	0.85 [0.46-1.02]	0.82
Recipient LISI	7.65 [4.67-8.85]	7.69 [4.19-10.3]	0.77
NK cell therapy	8 (29.6)	8 (29.6)	1.00
Operation time (min)	735 [670-797]	752 [657-815]	0.57
Bleeding volume (mL)	2,966 [2,100-5,040]	3,580 [2,150-4,700]	0.59
AFP (ng/mL)	13.4 [5.6-108]	6.8 [5-40.3]	0.30
DCP (mAU/mL)	27 [17-96]	34 [15-98]	0.71
Timor size (mm)	22 [14-28]	19 [10-25]	0.28
Tumor number	2 [1-4]	2 [1-4]	0.70
Vascular invasion, yes	6 (22.2)	4 (14.8)	0.48
Poorly differentiated, yes	2 (7.4)	2 (7.4)	1.00
**Donor factors**
Gender, male	11 (40.7)	22 (81.5)	<0.01
Age (years)	48 [33-54]	29 [25-34]	<0.01
BMI (kg/m^2^)	20.7 [19.7-21.9]	22.8 [20.4-24.4]	<0.01
Donor fibrosis-4 index	0.94 [0.62-1.21]	0.55 [0.41-0.76]	<0.01
Donor LISI	0.68 [−0.69-2.12]	−3.16 [−3.99-2.41]	<0.01

BMI, body mass index; GWRW, graft weight-recipient weight ratio: NK, natural killer; AFP, α-fetoprotein; DCP, des-γ carboxyprothrombin; LISI, liver immune status index

### Overall patient outcomes

**Figure 1.**
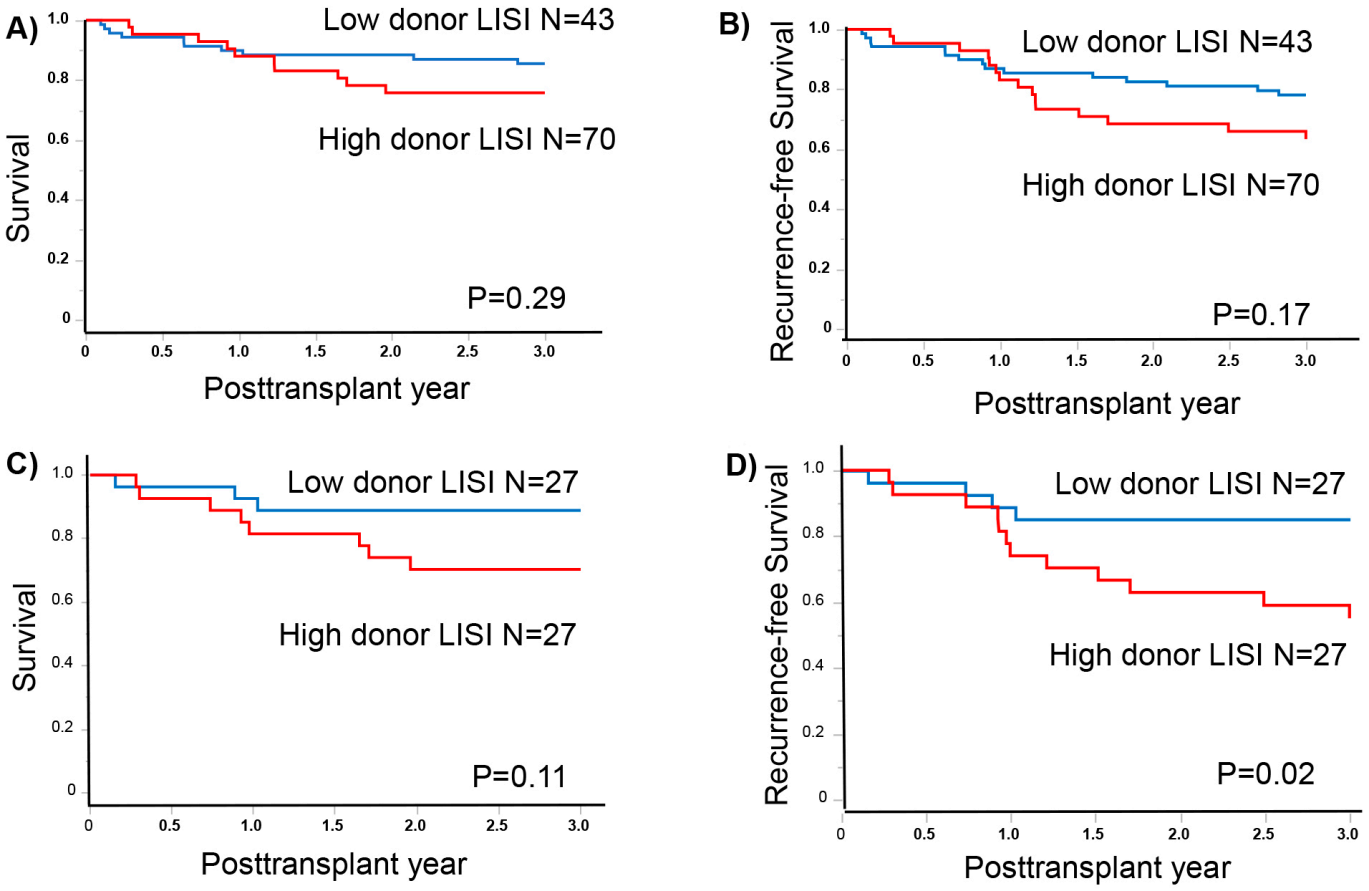
The outcomes before and after propensity matching (A) The 3-year survival before propensity matching (B) The 3-year recurrence-free survival (RFS) before propensity matching (C) The 3-year survival after propensity matching (D) The 3-year RFS after propensity matching.

The assessment of patient outcomes based on donor LISI grades demonstrated notable stratification in the OS and RFS metrics. Specifically, the observed 1- and 3-year OS rates were 89.9% and 85.5%, respectively, in patients in the low donor LISI group. Contrastingly, these rates were 88.0% and 75.8%, respectively, in patients in the high donor LISI group (p = 0.24; Figure 1A). Regarding RFS, the 1- and 3-year rates were 86.9% and 78.1% and 83.1% and 63.4% in the low and high donor LISI groups, respectively (p = 0.12; Figure 1B). After propensity score matching, the 1- and 3-year OS rates were 92.6% and 88.9% and 81.5% and 70.4% in the low (N = 27) and high (N = 33) donor LISI groups, respectively (p = 0.11; Figure 1C). Concurrently, the 1- and 3-year RFS rates were 88.9% and 85.2% and 74.1% and 55.1% in the low and high donor LISI groups, respectively. The RFS rate was significantly worse in the high donor LISI group than in the low donor LISI group (p = 0.02; Figure 1D). Among the recipients with NK cell therapy, the high donor LISI group had a significantly good prognosis in the 3-year OS and RFS (93.6% vs 70.0%, p = 0.03; and 87.1% vs 60.0%, p = 0.03, respectively).

### The influence of donor LISI on advanced HCC

**Figure 2.**
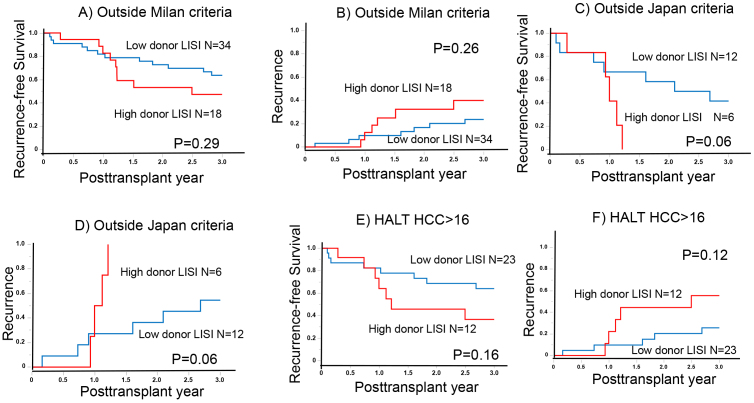
Outcomes among advanced hepatocellular carcinoma (HCC) (A) The recurrence-free survival (RFS) among outside the Milan criteria (B) The recurrence rate (RR) among outside the Milan criteria (C) RFS among outside the Japan criteria (D) RR among outside the Japan criteria (E) RFS among higher HALT-HCC (F) RR among higher HALT-HCC.

In the context of advanced HCC, the role of donor LISI becomes increasingly significant, particularly considering the known function of NK cells in targeting circulating tumor cells (CTCs) ^(11)^. Among patients outside the Milan criteria, the 1- and 3-year RFS rates were 82.1% and 63.8% and 82.6% and 47.2% in the low (N = 34) and high (N = 18) donor LISI groups, respectively (p = 0.29; Figure 2A). The corresponding RR for these groups were 9.8% and 23.7% and 12.5% and 40.0% (p = 0.26; Figure 2B).

Similarly, there were notable differences in RFS and RR among patients outside the Japan criteria. The 1- and 3-year RFS rates were 66.7% and 41.6% and 41.7% and 0% in the low (N = 12) and high (N = 6) donor LISI groups, respectively (p = 0.06; Figure 2C). The RRs at these intervals were 27.3% and 54.6% and 50.0% and 100% in the low and high donor LISI groups, respectively (p = 0.06; Figure 2D).

Furthermore, in advanced HCC cases with higher HALT-HCC scores (exceeding 16), the 1- and 3-year RFS rates were 82.4% and 64.1% and 64.2% and 36.7% in the low (N = 23) and high (N = 12) donor LISI groups, respectively (p = 0.16; Figure 2E). The corresponding 1- and 3-year RRs were 9.8% and 25.7% and 13.9% and 55.6% in the low and high donor LISI groups, respectively (p = 0.12; Figure 2F). These findings underscore the importance of donor LISI in influencing patient outcomes in LT, particularly in the context of advanced HCC.

### The influence of recipient LISI on advanced HCC

Subsequently, to elucidate the effect of recipient LISI on the clinical outcomes, a k-means cluster analysis was conducted. This analysis used both donor and recipient LISI values for stratification into three distinct cohorts: those characterized by high donor LISI (N = 35), high recipient LISI (N = 50), and a normal group (N = 28).

**Figure 3.**
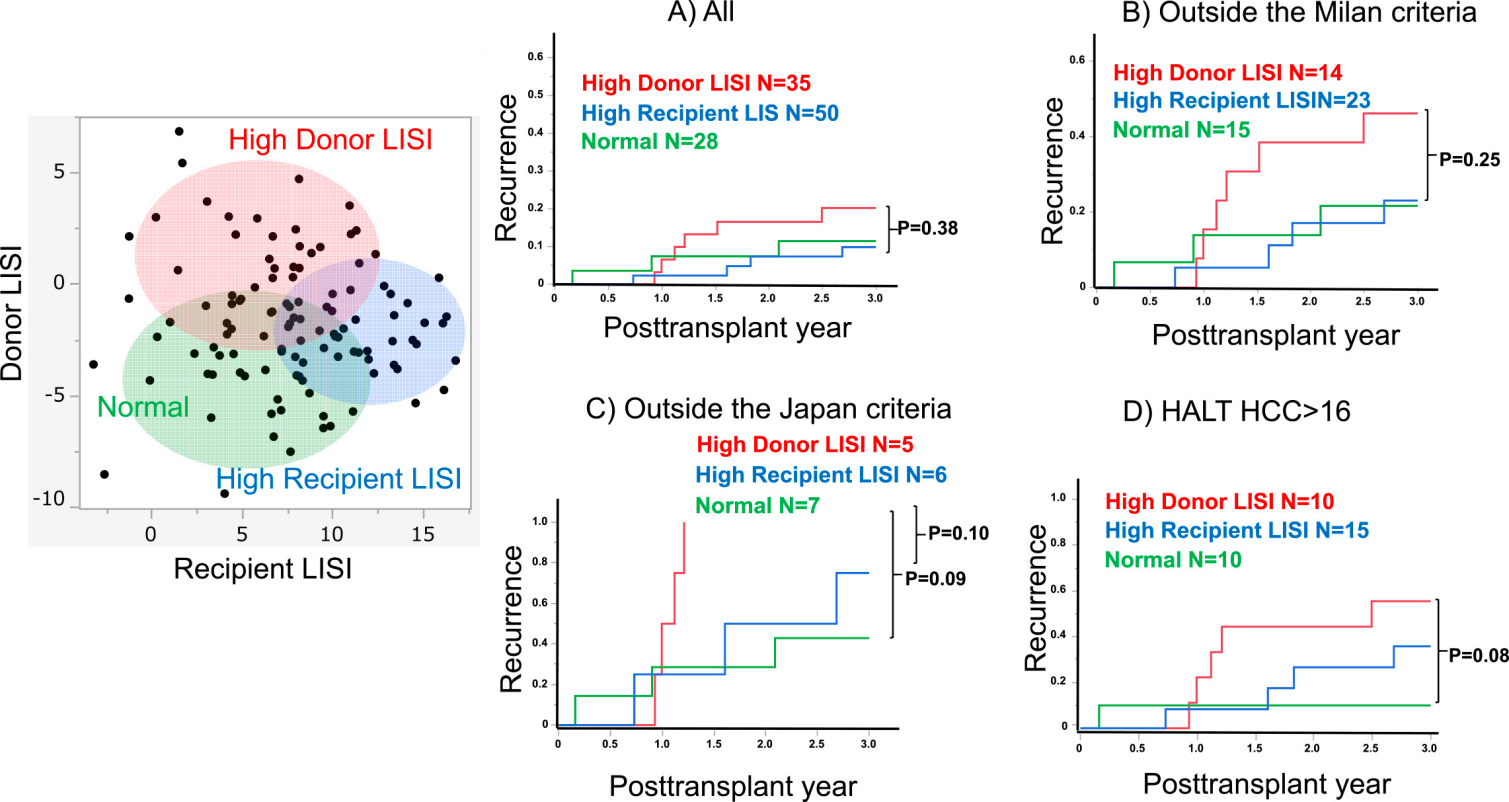
The outcomes according to the donor and recipient liver immune status index (LISI) (A) In all cases (B) Outside the Milan criteria (C) Outside the Japan criteria (D) Higher HALT-hepatocellular carcinoma (HCC) score.

As shown in Figure 3A, a comparative analysis across the three delineated groups revealed no statistically significant differences in RR among all cases. However, in cases of advanced HCC, including outside the Milan and Japan criteria and higher HALT-HCC score, both the high recipient LISI and normal groups exhibited analogous recurrence patterns. Contrarily, the high donor LISI group demonstrated an elevated RR in comparison with both the aforementioned groups, as further illustrated in Figure 3B, 3C, and 3D. It should be observed that the high donor LISI group showed a higher frequency of early recurrences approximately 1 year posttransplantation, particularly in cases of advanced HCC (Figure 3B, 3C, and 3D).

## Discussion

This investigation delineates the role of the donor LISI, computed using BMI, serum albumin levels, and the FIB-4 index, in augmenting the risk of early recurrence following LDLT for HCC. This is particularly evident in advanced HCC, which is associated with an increased likelihood of detecting CTCs. The findings bolster the hypothesis that NK cells target CTCs, thereby mitigating the risk of early recurrence. Previous research predominantly attributes post-LT recurrence in HCC to tumor characteristics ^(6)^; however, this study suggests that the antitumor milieu within the donor liver exerts a modest influence on early recurrence, specifically within a 3-year time frame. It was known that the antitumor immunity within the recipient liver was understood to be diminished ^(12)^. In this analysis, the recipient LISI appeared to have no discernible impact on early recurrence. As a noninvasive measure for assessing the antitumor capacity of liver NK cells, the LISI emerges as a pertinent index with potential applications in various oncological contexts, including LDLT for HCC. Consequently, the LISI could serve as a valuable biomarker across diverse oncological fields, especially in the realm of liver cancer.

NK cells are integral to the innate immune response, playing a pivotal role in tumor prevention ^(13)^. Their involvement in the detection and eradication of CTC is crucial for preventing recurrence ^(14)^. In LT, immunosuppressive agents are used beginning after LT to prevent acute rejection, thus suppressing T cell-based acquired immunity, and NK cells and other innate immunity are the main sources of antitumor immunity. A higher proportion of immature NK cells is observed in the liver, which are known for their elevated production of cytokines and TRAIL ^(15), (16), (17)^. Liver NK cells, predominantly comprising immature, are characterized by their expression of TRAIL, which they use to exert potent cytotoxic effects through the TRAIL-TRAIL receptor pathway ^(9), (12), (18), (19)^. This mechanism underscores the significant role of liver NK cells in maintaining intrahepatic immunity. Notably, single nucleotide polymorphisms in the TRAIL gene have been implicated in contributing to distant metastatic recurrence following HCC liver resection ^(20)^. The LISI score, which is an accurate predictor of TRAIL expression specifically in liver NK cells with interindividual variability, represents a valuable parameter for stratifying high-risk patients with vascular invasion following hepatic resection ^(7)^. Our results indicate that donor LISI, not recipient LISI, had a correlation with the 3-year RFS after LDLT for HCC. The LISI has demonstrated utility in cases of HCC involving liver resection with vascular invasion, as well as in HCC LT scenarios. However, its applicability extends beyond these contexts. LISI may also serve as a predictive tool for early prognosis in adoptive immunotherapy using liver NK cells. This suggests that the relevance of LISI is not confined to HCC management alone but has implications for a wider range of adoptive immunotherapy to predict effectiveness.

However, in Japan, the selection criteria for donor candidates in LDLT are notably stringent, thereby imposing substantial limitations on the practical application of the LISI in the donor selection process, particularly for LT for HCC. This restrictive context for donor candidacy significantly complicates the use of the LISI score in this setting. Indeed, the high donor LISI defined in this study accounts for 38% of the total. Consequently, future research endeavors exploring the relevance of LISI in the context of DDLT for HCC in regions such as Europe and the United States―where a broader spectrum of donor options is available―will be instrumental. It is essential to recognize that significant disparities exist between deceased-donor grafts commonly encountered in Europe and the United States and those of living donors in Japan. These differences encompass several factors such as the prevalence of hepatitis virus, prolonged ischemia time, racial demographics, and BMI. Consequently, there is a pressing need to develop a modified LISI that is attuned to these new population characteristics. Such a tailored approach would ensure that the LISI is appropriately calibrated to the specificities of different donor profiles and transplantation practices across various geographic and demographic contexts. Such studies are anticipated to enhance our comprehension of liver antitumor immunity, with a specific focus on the role of NK cells. This international perspective and comparative approach could potentially unravel new dimensions of understanding in LT immunology.

This investigation, while offering valuable insights, is constrained by several notable limitations that merit consideration. First, as a single-center study examining experiences with LDLT, it is based on a relatively small patient cohort. This limitation necessitates caution in generalizing the findings, emphasizing the need for validation through larger, multicenter studies to confirm the applicability of the donor LISI. Additionally, the LISI, derived from assessments of living donors’ typically healthy livers, may not directly translate to patients with compromised hepatic function. This discrepancy underlines the importance of cautious interpretation and potential modification of the LISI in contexts involving varying degrees of liver pathology in patients.

Furthermore, approximately 37% of the patient cohort received adoptive immunotherapy, a treatment whose safety has been established in phase I clinical trials. However, its efficacy in enhancing therapeutic outcomes, especially in the context of LDLT for HCC, is yet to be fully determined. The absence of definitive evidence regarding its therapeutic impact necessitates a more thorough examination in future research.

### Conclusions

This study concludes that the donor LISI plays a significant role in increasing the risk of early RR after LDLT for HCC.

## Article Information

This article is based on the study, which received the Medical Research Encouragement Prize of The Japan Medical Association in 2023.

### Conflicts of Interest

None

### Sources of Funding

This work was supported in part by JSPS KAKENHI grant numbers JP22K16534, 22K16535, and 23H02981 and AMED grant number 23fk0210108. This research was awarded the Japan Medical Association Award for Encouragement of Medical Research.

### Acknowledgement

We thank Editage (www.editage.jp) for the English language review.

### Author Contributions

YI and MO conceived and designed the study. YI, MO, SS, IC, BT, KI, KS, NR, RY, and HS acquired the data and did all the experiments. YI and MO analyzed and interpreted the data. YI and MO drafted the manuscript. YI, MO, NR, RY, HS, SK, HT, KI, TK, YT, MA, KS, and HO critically revised the article. YI, MO, SS, IC, BT, KI, KS, NR, RY, HS, SK, HT, KI, TK, YT, MA, KS, and HO approved the final version of the manuscript to be published.

### Approval by Institutional Review Board (IRB)

The study protocol was approved by the ethics committee of our hospital (E-1937).

### Informed Consent

The study conforms to the provisions of the Declaration of Helsinki. The need for written informed consent was waived because of the retrospective nature of the study. The opt-out method to obtain patient consent was used at all institutions.

## References

[1] Rumgay H, Arnold M, Ferlay J, et al. Global burden of primary liver cancer in 2020 and predictions to 2040. J Hepatol. 2022;77(6):1598-606. 36208844 10.1016/j.jhep.2022.08.021 PMC9670241

[2] Fernandez-Sevilla E, Allard MA, Selten J, et al. Recurrence of hepatocellular carcinoma after liver transplantation: is there a place for resection? Liver Transpl. 2017;23(4):440-7. 28187493 10.1002/lt.24742

[3] European Association for the Study of the Liver. EASL clinical practice guidelines: management of hepatocellular carcinoma. J Hepatol. 2018;69(1):182-236. 29628281 10.1016/j.jhep.2018.03.019

[4] Regalia E, Fassati LR, Valente U, et al. Pattern and management of recurrent hepatocellular carcinoma after liver transplantation. J Hepato-Bil Pancreat Surg. 1998;5(1):29-34. 10.1007/pl00009947 9683751

[5] Shinkawa H, Tanaka S, Takemura S, et al. Prognostic value of expanded liver transplantation criteria-the 5-5-500 rule-in patients with hepatic resection for intermediate-stage hepatocellular carcinoma. J Hepato-Bil Pancreat Sci. 2020;27(10):682-9. 10.1002/jhbp.792 32578373

[6] Sasaki K, Firl DJ, Hashimoto K, et al. Development and validation of the HALT-HCC score to predict mortality in liver transplant recipients with hepatocellular carcinoma: a retrospective cohort analysis. Lancet Gastroenterol Hepatol. 2017;2(8):595-603. 28546007 10.1016/S2468-1253(17)30106-1

[7] Imaoka Y, Ohira M, Chogahara I, et al. Impact of a new liver immune status index among patients with hepatocellular carcinoma after initial hepatectomy. Ann Gastroenterol Surg. 2023;7(6):987-96. 37927921 10.1002/ags3.12702 PMC10623950

[8] Sterling RK, Lissen E, Clumeck N, et al. Development of a simple noninvasive index to predict significant fibrosis in patients with HIV/HCV coinfection. Hepatology. 2006;43(6):1317-25. 16729309 10.1002/hep.21178

[9] Ohira M, Ishiyama K, Tanaka Y, et al. Adoptive immunotherapy with liver allograft-derived lymphocytes induces anti-HCV activity after liver transplantation in humans and humanized mice. J Clin Invest. 2009;119(11):3226-35. 19805910 10.1172/JCI38374 PMC2769186

[10] Ohira M, Imaoka Y, Sato K, et al. A phase I/II study of adoptive immunotherapy using donor liver graft-derived natural killer cells to prevent bloodstream infection after liver transplantation: a study protocol. Transl Med Commun. 2022;7(1):19.

[11] Carrette F, Vivier E. NKG2A blocks the anti-metastatic functions of natural killer cells. Cancer Cell. 2023;41(2):232-4. 36787695 10.1016/j.ccell.2023.01.008

[12] Ishiyama K, Ohdan H, Ohira M, et al. Difference in cytotoxicity against hepatocellular carcinoma between liver and periphery natural killer cells in humans. Hepatology. 2006;43(2):362-72. 16440347 10.1002/hep.21035

[13] Trinchieri G. Biology of natural killer cells. Adv Immunol. 1989;47:187-376. 2683611 10.1016/S0065-2776(08)60664-1 PMC7131425

[14] Dianat-Moghadam H, Mahari A, Heidarifard M, et al. NK cells-directed therapies target circulating tumor cells and metastasis. Cancer Lett. 2021;497:41-53. 32987138 10.1016/j.canlet.2020.09.021

[15] Jacobs R, Hintzen G, Kemper A, et al. CD56bright cells differ in their KIR repertoire and cytotoxic features from CD56dim NK cells. Eur J Immunol. 2001;31(10):3121-7. 11592089 10.1002/1521-4141(2001010)31:10<3121::aid-immu3121>3.0.co;2-4

[16] Krueger PD, Lassen MG, Qiao H, et al. Regulation of NK cell repertoire and function in the liver. Crit Rev Immunol. 2011;31(1):43-52. 21395510 10.1615/critrevimmunol.v31.i1.40 PMC3163300

[17] Saparbay J, Tanaka Y, Tanimine N, et al. Everolimus enhances TRAIL-mediated anti-tumor activity of liver resident natural killer cells in mice. Transpl Int. 2020;33(2):229-43. 31560810 10.1111/tri.13536

[18] Takeda K, Hayakawa Y, Smyth MJ, et al. Involvement of tumor necrosis factor-related apoptosis-inducing ligand in surveillance of tumor metastasis by liver natural killer cells. Nat Med. 2001;7(1):94-100. 11135622 10.1038/83416

[19] Ohira M, Ohdan H, Mitsuta H, et al. Adoptive transfer of TRAIL-expressing natural killer cells prevents recurrence of hepatocellular carcinoma after partial hepatectomy. Transplantation. 2006;82(12):1712-9. 17198265 10.1097/01.tp.0000250935.41034.2d

[20] Imaoka Y, Ohira M, Yano T, et al. Polymorphisms in TRAIL predict long-term survival and extrahepatic recurrence following initial hepatectomy for hepatocellular carcinoma. J Hepato-Bil Pancreat Sci. 2018;25(8):370-6. 10.1002/jhbp.573 30051596

